# Apparent “double wall sign” in emphysematous bullae of the lung

**DOI:** 10.11604/pamj.2026.53.154.43815

**Published:** 2026-04-13

**Authors:** Lokesh Devalla

**Affiliations:** 1 Department of Respiratory Medicine, Jawaharlal Nehru Medical College, Datta Meghe Institute of Higher Education and Research, Sawangi (Meghe), Wardha, Maharashtra, India

**Keywords:** Cough, dyspnea, cavity, emphysema, pneumothorax

## Image in medicine

A 52-year-old female patient came to the hospital with complaints of progressive breathlessness and dry cough on and off for 6 months, relieved with medications. High-Resolution Computed Tomography (HRCT) of the thorax showed a large thick-walled cavity with an air-fluid level in the right hemithorax and bilateral pan-acinar, paraseptal emphysematous changes with adjacent consolidation and ground-glass opacities termed as bullae. A bulla is an air-containing structure bigger than 1 cm in diameter that develops inside the lung parenchyma as a result of disruption, dilation, and convergence of airspaces distal to terminal bronchioles. Massive bullous emphysema, when causing progressive dyspnea, is called “vanishing-lung syndrome.” They are avascular radiolucent patches with curvilinear walls less than 1 mm in thickness, so challenging to identify radiologically from pneumothorax. A computed tomography (CT) scan is more sensitive for a precise evaluation, particularly when the bullae are veiled. The clinical image depicts a valuable “double-wall sign” that helps identify pneumothorax from adjacent giant bullae. When air is seen on either side of the bulla wall and parallel to the chest wall, the sign becomes obvious, and the lack of this sign reduces the probability of pneumothorax. A potential pitfall is the false visualisation of a double-wall sign when two bullae are adjacent to one another, sometimes simulating a pneumothorax and causing an apparent double-wall sign. However, a detailed study will show that the pleural gap is airless and the bulla wall is perpendicular to the chest wall. Hence, it is critical to distinguish between bullae and pneumothorax precisely to prevent iatrogenic pneumothorax from needless chest tube insertion.

**Figure 1:**
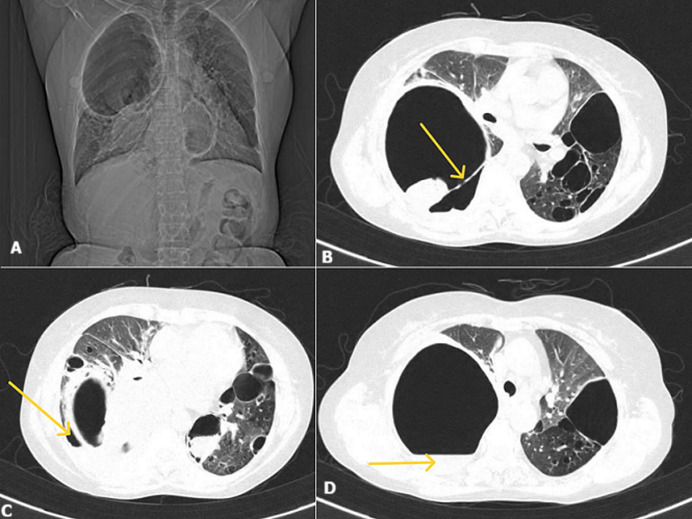
A) chest X-ray showing large right-sided bulla; B) high-resolution computed tomography thorax showing thick wall indicated with arrow; C) sign of double wall (arrow); D) water fluid level indicated with arrow

